# Determination of drug sensitivity in patient derived models of breast cancer by multiparametric quantitative phase imaging

**DOI:** 10.1186/s12885-026-16752-2

**Published:** 2026-08-12

**Authors:** Edward R. Polanco, Tarek E. Moustafa, Ozlen Balcioglu, Sandra D. Scherer, Emilio Cortes-Sanchez, Sophia M. Remick, Benjamin T. Spike, Alana L. Welm, Bryan E. Welm, Philip S. Bernard, Thomas A. Zangle

**Affiliations:** 1 Department of Chemical Engineering, University of Utah, Salt Lake City, UT USA; 2 Huntsman Cancer Institute, University of Utah, Salt Lake City, UT USA; 3 Department of Oncological Sciences, University of Utah, Salt Lake City, UT USA; 4 Department of Surgery, University of Utah, Salt Lake City, UT USA; 5 Department of Pathology, University of Utah, Salt Lake City, UT USA; 6 ARUP Institute for Clinical and Experimental Pathology, Salt Lake City, UT USA

**Keywords:** Quantitative phase imaging, Functional precision medicine, Breast cancer, *In vitro* models

## Abstract

**Background:**

Functional precision oncology aims to guide therapy selection by directly measuring drug sensitivity in patient-derived cancer samples. However, most existing functional assays require prolonged cell expansion limiting their feasibility for time-sensitive clinical decision-making. We evaluated whether multiparametric quantitative phase imaging (mQPI), a label-free single-cell imaging approach, could enable rapid assessment of therapeutic response in patient-derived breast cancer models.

**Methods:**

We applied mQPI to cells derived from patient-derived xenograft organoid (PDXO) models and viably cryopreserved primary breast cancer samples. Quantitative phase imaging was used to extract multiple orthogonal biophysical parameters describing cellular growth and response dynamics following drug exposure. Drug sensitivity, intrapatient heterogeneity, and resistance-associated phenotypes were quantified and compared across models.

**Results:**

mQPI resolved distinct drug response profiles among cells derived from different anatomical sites within the same patient and revealed heterogeneous response dynamics in models of acquired therapeutic resistance. Importantly, drug responses were detected in a high-purity cryopreserved patient sample immediately after thawing, whereas a more heterogeneous sample required a short-term (2-week) expansion to enrich the viable tumor population before a response could be resolved, indicating that sample composition determines whether a direct-from-thaw or short-term expansion workflow is required. Across sample types, mQPI enabled robust single-cell measurements without the need for labeling or extensive culture.

**Conclusions:**

These findings establish mQPI as a rapid, label-free functional assay capable of quantifying therapeutic response and heterogeneity in patient-derived breast cancer samples. By reducing assay time and material requirements while preserving single-cell resolution, mQPI has the potential to complement genomic profiling and advance the clinical translation of functional precision oncology.

**Supplementary Information:**

The online version contains supplementary material available at https://doi.org/10.1186/s12885-026-16752-2.

## Background

Precision oncology uses genomics and comprehensive sequencing to identify molecular alterations in tumors for targeted therapy [1, 2]. While a molecular approach has improved survival for patients with some cancer types, many variants/mutations lack evidence of clinical utility and the emergence of resistance to targeted drugs is common [3, 4]. These challenges have led to an emerging discipline of precision oncology that uses patient derived models of cancer (PDMC) to determine a functional drug response for guiding patient therapy [5–8] For example, drug responses in human breast cancers grown in patient derived xenograft (PDX) models show high agreement to responses in the patient. However, these PDX models can require up to 6 months of tumor expansion and can then only screen a few therapies. Thus, these methods are not amenable to clinical testing due to high cost, slow turnaround time, and low throughput.

To reduce reliance on in vivo animal testing and expand the repertoire of clinically relevant tumor models, functional oncology has increasingly used ex vivo systems that capture therapeutic response. Patient-derived xenograft organoids (PDXOs) [9] retain salient genetic, phenotypic, and drug response features of originating tumors and have enabled high-throughput drug sensitivity screening in multiple cancer types, including breast cancer. In parallel, advanced patient-derived functional profiling platforms have emerged with a focus on timelines compatible with clinical decision making. Droplet microfluidic micro-organospheres (MOSs) can be generated from small patient biopsies and used to assess drug sensitivity within ~ 14 days, recapitulating clinical response patterns and enabling evaluation of both targeted agents and immunotherapies [10]. Similarly, molecularly guided functional assays such as dynamic BH3 profiling measure early apoptotic priming after drug exposure and have demonstrated predictive value for therapeutic response [11, 12]. In traditional patient derived organoid (PDO) or PDXO workflows, drug sensitivity is often quantified using endpoint viability assays like CellTiter-Glo (CTG) to derive EC50 values, but these bulk metabolic readouts do not resolve single-cell heterogeneity or temporal response dynamics that emerge during treatment. Single cell measurement of mass after drug exposure via microchannel resonators is another promising approach [13, 14], though this approach has an inherent tradeoff between longitudinal measurements and throughput. Together, these functional precision oncology efforts underscore a central challenge: developing rapid, scalable assays that preserve heterogeneity and dynamic behavior in patient-derived material while producing clinically actionable insights on therapeutic sensitivity.

One emerging approach for studying the temporal dynamics of cell response to therapy is quantitative phase imaging (QPI) [15], a quantitative microscopy technique that measures changes in the optical path length as light passes through cellular material [16]. This phase shift of light is proportional to cell dry mass, thus, allowing QPI to measure cell growth and death of single cells as the accumulation and loss of mass over time respectively [17, 18]. QPI is label-free, facilitating the study of live cells without disrupting cell activity [19]. This is especially important for application to clinical samples which may have limited number and that may be sensitive to labeling approaches. Initial QPI systems were based on interferometry [20, 21] while later techniques include tomography [22], holography [23], and wave front sensing [24]. Each of these techniques comes with varying levels of precision and accuracy [25] as well as system specific optical complexity, references, alignment challenges and computational requirements. For example, a standard brightfield microscope can be used with computational techniques that implement the transport of intensity equation to compute phase shifts from out of focus images [26, 27]. Cross-grating wavefront microscopy (CGM)/quadriwave lateral shearing microscopy (QLSI), a wavefront sensing approach, can be performed with commercially available cameras on standard research microscopes [28–30]. Fourier ptychography, which folds images captured under a wide range of illumination to capture phase shifts at resolution beyond the diffraction limit [31, 32], or digital holography [33–35], can be performed on custom or commercial purpose-built systems. Finally, differential phase contrast (DPC) [36], a computational microscopy approach, combines four brightfield images captured using an LED array as a light source on an otherwise standard microscope to compute phase [36, 37]. Of these, DPC, in particular, offers a robust platform that is well-suited to high-throughput functional assays.

QPI has been used to quantify growth and other cellular behaviors in a variety of settings. For example, previous work used QPI to examine biomass partitioning during cell division [17], cell death [38–40], and transport of bulk material inside a cell [41, 42]. QPI has also been used to study the mechanical properties of cells such as the shear modulus [43] and viscoelasticity [44, 45]. The dynamics of neuron growth and branching has also been previously studied with QPI [46–48]. QPI can also be used to monitor disease progression in terms of changes in tissue structure [49–51]. With the aid of machine learning approaches, QPI has been used to generate synthetic H&E staining [52], and to track organelles [53].

A key application of QPI involves the study cell behavior in response to perturbations such as drug response [54–56]. The high precision of QPI enables rapid assessment of response to drug treatment [57] or in bioprinted organoids [58]. In our previous work on breast cancer cell lines, we developed several response parameters that leverage unique capabilities of QPI [55]. Since timelapse QPI measures changes in mass with single cell resolution, we were able to determine the growth rate of individual cells and heterogeneity in populations as they change under therapeutic challenge. Accordingly, we were also able to establish EC_50_ values by QPI, i.e. the relative concentration at which half the maximal response to therapy occurs and these values were highly concordant with those obtained by CTG [55]. Furthermore, QPI enables depth of response (DoR) measurements as the difference between the asymptotes along a dose response curve normalized by the baseline growth rate of the control. QPI can also leverage time-series data to study the temporal dynamics of cell behavior, such as the time required to elicit a drug response, which we describe here as time of response (ToR). Taken together, these various parameters allow QPI to provide a multiparametric tool that builds a more robust picture of how cancer cells are responding to therapy.

Here, we apply QPI as a multiparametric approach for understanding how PDXO samples respond to cancer therapies. We demonstrate that QPI can identify the difference in response between PDXO models derived from different tumor sites within the same patient. We also show that QPI can be used to study heterogeneous samples and the emergence of resistant subpopulations. We also demonstrate the application of multiparametric QPI to CD45 depleted tumor cells in direct-from-thaw patient samples, and short-term expanded patient samples, which we show greatly enriches the tumor content of the sample. Toward a practical solution for precision oncology, this approach can provide empirical drug response data for solid tumors on a patient-by-patient basis with lower time and material overhead than existing methods.

## Methods

### 96-well plate preparation

10x plate coating solution was prepared by diluting 25,000 MW branched polyethylenimine (PEI, Millipore Sigma, USA) at 0.1% in borate buffer (pH 8.4). Immediately prior to plating, 10x PEI was then diluted in borate buffer to reach 0.01% working concentration. 96-well plates (Eppendorf, Germany) were treated with 700 µL of 0.01% PEI per well for 4–12 h then washed twice with autoclaved deionized water. Plates were either used immediately or stored up to two weeks at 4 °C.

### PDXO model culture

The PDXO models were established in the Welm lab at the Huntsman Cancer Institute at the University of Utah and were cultured as described previously [59] for 1–2 passages (2–8 weeks) post thaw prior to plating for QPI. 18–24 h prior to imaging via QPI, PDXOs were dissociated into single cell suspension as described previously [59] and plated at a density of 5,000 cells per well using PDXO media [59]. The moat in the 96-well plate was filled with sterile, deionized water to minimize evaporation (8 mL inner moat and 5 mL outer moat).

### Primary cell culture

Patient samples were thawed, washed and resuspended at a maximum of 1 × 10^8^ cells/mL in cold PBS containing 2% fetal bovine serum (FBS) and 1 mM EDTA. Cells were immune cell depleted by anti-using EasySep CD45 antibody Depletion Kit II (StemCell, Cat # 17898) (1x for patient 1, 2x for patient 2). Cells were then pelleted and resuspended in Clevers media [60] then plated at 12,000–15,000 cells per well in PEI coated 96-well plates, or plated for 2-week expansion with one intermediate media change, then plated for QPI. Fresh media was provided immediately prior to treatment and imaging by QPI.

### Drug dosing

Cells were dosed (Table ST2, Selleck Chemicals, USA) 3 h prior to the start of imaging by adding 100 µL at 2x the dosing concentration diluted in media to wells containing 100 µL of media. Vehicle (DMSO or ethanol) concentrations were maintained at 0.1% across all wells to account for potential vehicle toxicity.

### Quantitative phase imaging

QPI was performed using a custom DPC QPI microscope described and characterized previously [55]. The microscope is optimized to rapidly acquire data from 9 locations in each well of a 96-well plate (864 total locations) every 20 min. Experiments were performed in triplicate. The microscope uses an LED array for illumination (Adafruit, New York, USA) to rapidly switch between half circle illumination [36, 37, 61], a 10x Olympus Plan N objective lens (Olympus, Japan) with a numerical aperture of 0.25, a tube lens, and a Grasshopper3 machine vision monochromatic camera (Teledyne-FLIR, Oregon, USA) with an exposure time of 50 ms and gain of 25 dB. An MLS203 high-speed stage (Thorlabs, New Jersey, USA) is used for stage motion. The microscope is compact enough to operate inside of a tissue culture incubator to maintain cell culture conditions at 37 °C, 5% CO_2_, and 95% humidity. Raw DPC intensity images were processed for quantitative phase using Tikhonov regularization [37, 61] with a regularization parameter of 1 × 10^− 3^.

### Image processing

Image processing was completed using methods and code as described previously [30] (https://github.com/Zangle-Lab/MultiparametricQPI). Briefly, cells were segmented by Sobel filtering to find edges, followed by morphological processing to dilate segmented cell areas. Cell mass was computed using a specific refractive increment of 1.8 × 10^− 4^ m^3^/kg [62]. Cell tracking was performed by minimizing distance between paired cells [63] in x, y, position as well as mass.

### Growth rate processing

Specific growth rates (SGRs) were computed by least squares linear fitting to tracks longer than 20 frames after applying a median filter of size 5 frames. SGR was computed as the slope of this linear fit divided by the initial projected mass at the start time of the track. SGR data for each condition were averaged and used for Hill curve fitting:

$$\begin{aligned}SGR&={E}_{max}+\left({E}_{0}-{E}_{max}\right)\\&/\left[1+{\left(C/{EC}_{50}\right)}^{HS}\right]\end{aligned}$$

where *E*_*max*_ is the upper asymptote, *E*_*0*_ is the lower asymptote, *C* is drug concentration, *EC*_*50*_ is the inflection point of the Hill curve, and *HS* is the Hill slope. Depth of response (DoR) was then defined as:

$$DoR=\left({E}_{0}-{E}_{max}\right)/{E}_{0}$$

### Time of response calculation

To compute time of response (ToR), cell mass data were binned into 12 h windows centered on time points at 2 h increments. Slope was extracted from the model and normalized by the initial mass predicted by the linear fit, as in overall SGR calculations. Hellinger distance between two populations was then computed from the probability density functions of SGRs of those populations [64]. The Hellinger distance threshold for response was defined as the maximum Hellinger distance between the two on-plate controls for each experiment. For each drug treatment, we measured the Hellinger distance at each time point between the drug treated distribution and the control distribution and fit an exponential model to these data. ToR is defined as the time when the curve fit to the measured Hellinger distance crosses the threshold determined from control data.

### Gaussian mixture analysis

Mean SGR values at each time point were binned into probability histograms and smoothed using kernel density estimation (KDE) with a bandwidth of 0.0025 h^− 1^. KDE curves were then fit to a sum of two gaussians, or gaussian mixture model:

$$\begin{aligned}{y}_{mix}&=A*f*\mathrm{exp}\left(-{\left(\frac{x-{\mu}_{s}}{{\sigma}_{s}}\right)}^{2}\right)\\&+A*\left(1-f\right)\mathrm{*exp}\left(-{\left(\frac{x-{\mu}_{r}}{{\sigma}_{r}}\right)}^{2}\right)\end{aligned}$$

with *f* representing the fraction of the overall population that is sensitive and *A* the amplitude of the Gaussian distribution. *µ* and *σ* indicate the mean and standard deviation of the two Gaussian distributions, where subscripts indicate responsive, *r*, or non-responsive, *n*, populations. Initial fitting parameters for the first time point were estimated from the KDE amplitude (*A*_*KDE*_) the median (*m*_*all*_) and the standard deviation (*σ*_*all*_) of the overall population: *A*_*0*_= *A*_*KDE*_; *µ*_*r,0*_ = *m*_*all*_ - *σ*_*all*_/2; *µ*_*n,0*_ = *m*_*all*_ + *σ*_*all*_/2; *σ*_*t,0*_ = *σ*_*n,0*_ = *σ*_*all*_. The resulting parameters from each fit were then used as initial guesses for the next time point.

### Statistics

Group means were compared using a two-tailed, unpaired Welch’s t-test in MATLAB (RRID: SCR_001622). Variances between fits were compared using MATLAB’s ftest function, which performs a two-tailed F-test. Significance was set at *p* < 0.05. Lin’s concordance coefficient was used to measure the concordance between variables [65].

## Results

### Multiparametric QPI characterizes drug response of PDXO models

**Fig. 1:**
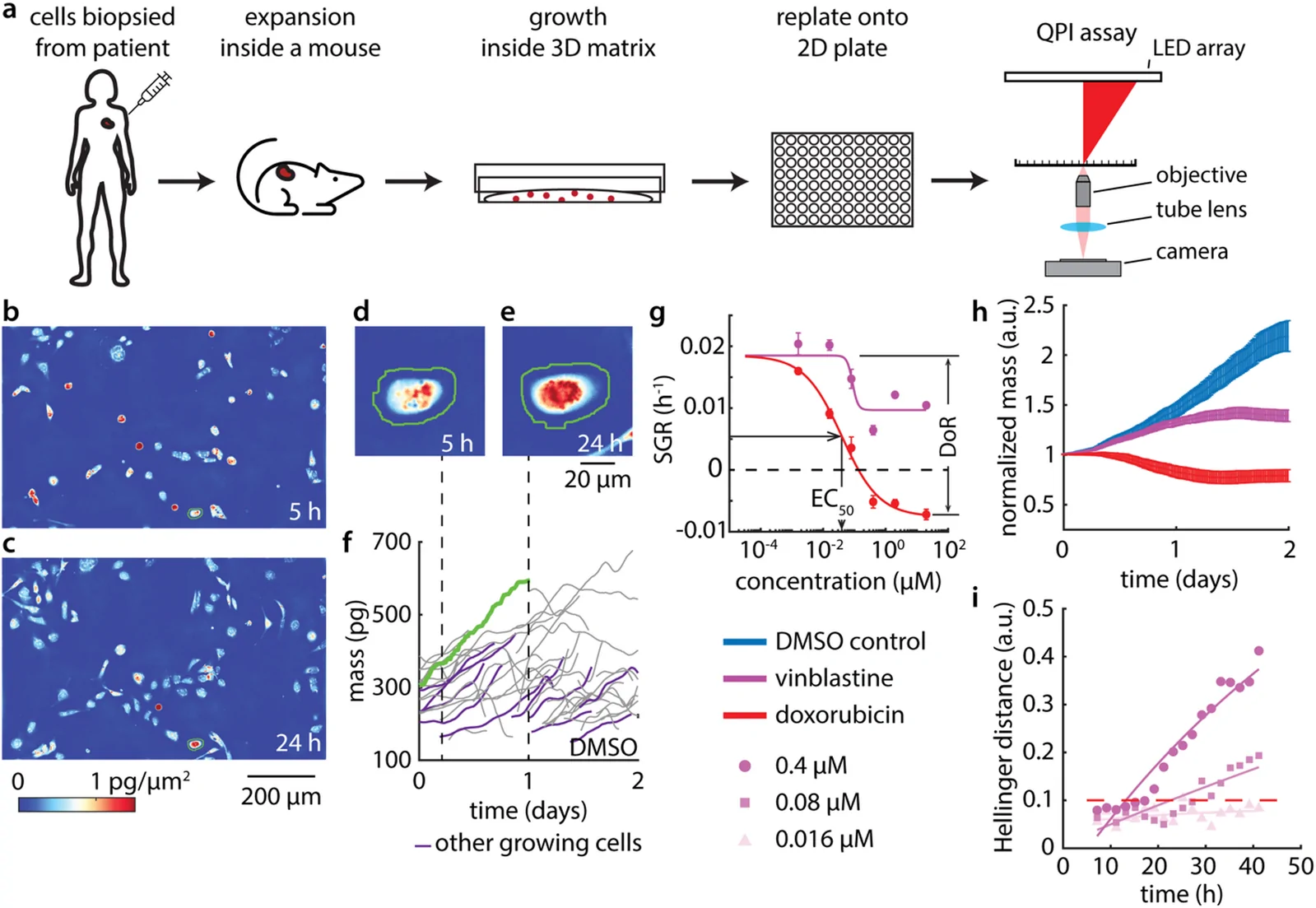
Multiparametric QPI characterizes functional response of PDXO model HCI-037 to cancer therapy. **a** Experiment workflow. **b**-**e** Images of DMSO (0.1%) treated HCI-037 PDXO cells in 2D culture at (**b**) 5 h and (**c**) 24 h. An individual HCI-037 PDXO cell at (**d**) 5 h and (**e**) 24 h. **f** Dry mass vs. time traces for the cell in (**d**-**e**) (green line) and other proximal cells (purple). **g** Dose response plot of HCI-037 for doxorubicin and vinblastine treatments from all individual cell tracks at each drug concentration. **h** Normalized mass shows the temporal dynamics of HCI-037 response to doxorubicin and vinblastine at the EC50, showcasing a drug dependent response. **i** Hellinger distance plot for 0.4, 0.08 and 0.016 µM vinblastine

We used mQPI to assess how drug treatment affects mass and growth rate of breast cancer cells from PDXO models at single cell resolution (Fig. 1). Short term two-dimensional (2D) cultures were established from PDXO models by dissociating organoids and seeding on polyethylenimine (PEI)-coated 96 well plates (Fig. 1a, S1). PEI, a positively charged polymer used for plating neural cell cultures [66], was found to be a suitable substrate for PDXO breast cancer cell growth and QPI imaging (Fig. S2). The PDXO models were previously established at the Huntsman Cancer Institute at the University of Utah to create a bank of PDXOs representing important clinical sub-types including triple negative breast cancer (TNBC), metastatic cancer, and treatment resistant cancers (Table ST1) [9, 59, 67]. Eighteen to twenty-four hours after plating in 2D, cultures were dosed with 5 different drug treatments in 6-point dose curves, and mQPI data was collected for 48 h (Fig. 1b-i, S1) (Fig. S3, Tables ST2, ST3).

We imaged cells for 48 h using an automatic segmentation algorithm to identify individual cells (Fig. 1b, c). The mass of each cell was determined by summing mass over the segmented area (Fig. 1d, e). Plots of mass vs. time reveal single cell growth rates (Fig. 1f), which we fit to a linear model by least-squares regression. Specific growth rate (SGR) was computed for each cell by normalizing the slope of this line by initial mass to account for the effect of cell size on growth rate [55, 68]. We then fit SGRs of each drug as a function of dose to a sigmoidal dose response curve described by a 3-parameter Hill model to extract dose response measurements, EC_50_ and depth of response (DoR) (Fig. 1g). Plots of total cell mass over time, normalized by initial mass, show the drug dependent nature of the population average temporal growth response at EC_50_. For example, some drugs, such as doxorubicin, show a large decrease in mass, starting at t = 0 h, while others, such as vinblastine, show a smaller effect on mass, with separation from control delayed to later timepoints (Fig. 1h). Each PDXO model was tested in triplicate against each drug dose. We observed low variation in control growth rate between biological replicates, indicating the repeatability of the QPI assay (Fig. S4).

Finally, to capture the time-dependence of drug effects, we compare the distribution of single-cell SGRs within each time window following treatment. For each drug-concentration pair, the difference between treatment and control was quantified using Hellinger distance [13] and fit to a single exponential [55]. Time of response (ToR) is then the time at which the fitted line crosses a certain threshold determined by comparison to the maximum variation observed over time in control wells (Fig. S4). ToR reveals concentration dependent temporal dynamics. For example, HCI-037 treated with increasing concentration of vinblastine show progressively faster ToR (Fig. 1i).

### Multiparametric QPI characterizes differential growth behavior between PDXO models from various sites of origin

**Fig. 2:**
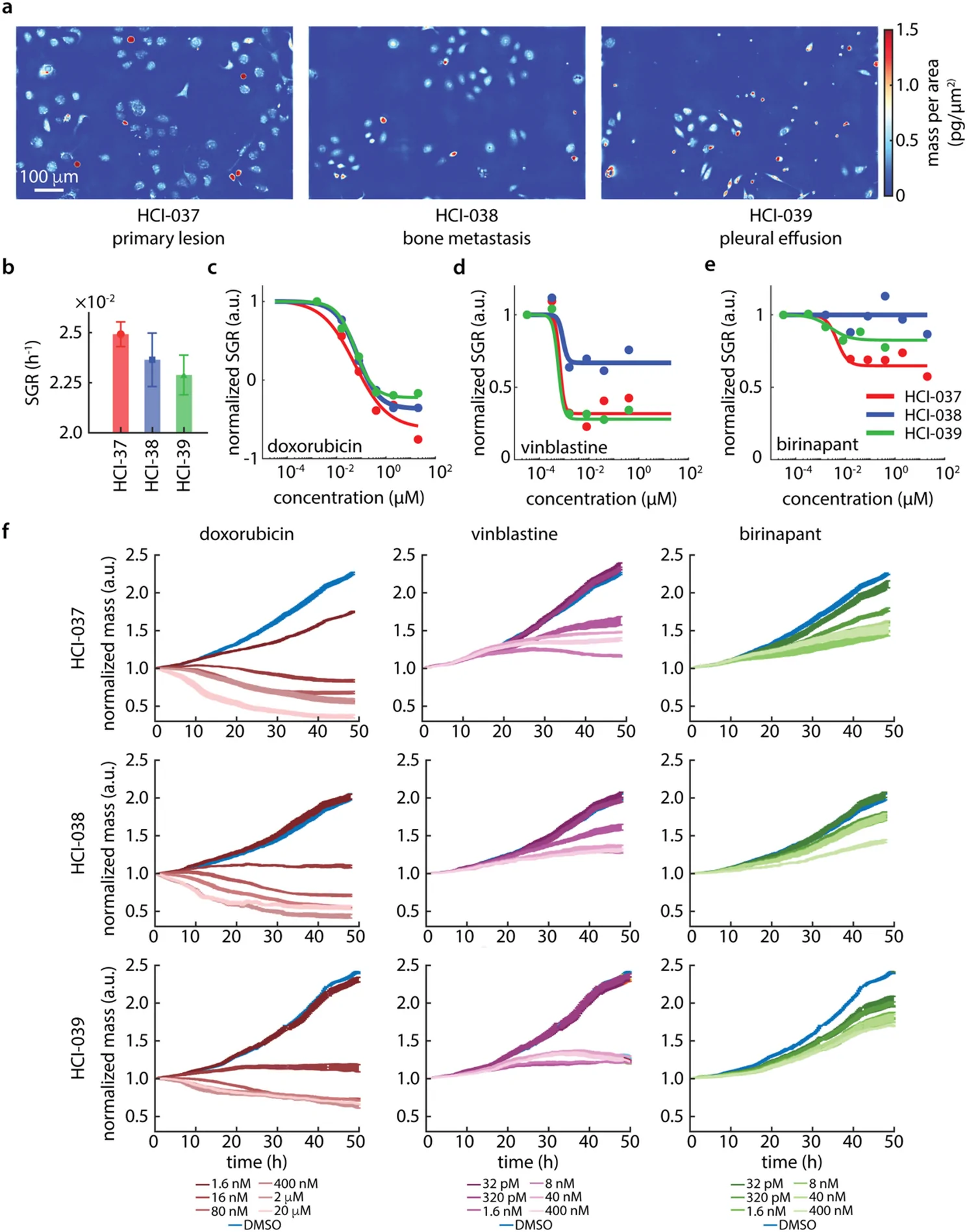
Multiparametric QPI shows differential response in tumor cells from different sites of origin. **a** Representative QPI images of three PDXO models from the same patient, HCI-037 (primary), HCI-038 (bone metastasis), and HCI-039 (pleural effusion). **b** Population average SGR for each model (0.1% DMSO). **c**-**e** Dose response plots for each model with (**c**) doxorubicin, (**d**) vinblastine, and (**e**) birinapant treatment. Colors indicate model (red: HCI-037, blue: HCI-038, green: HCI-039). **f** Longitudinal measurement of mass normalized to initial masses for the same drugs as in (**c**-**e**) at varying dose (darker: lower dose). Error bars show SEM

We used multiparametric QPI to characterize the difference in response from three PDXO models derived from a single patient biopsied at different timepoints, locations, and with different intervening treatments. These include the primary tumor (HCI-037), bone metastasis (HCI-038), and pleural effusion (HCI-039) [9]. These different PDXO models have distinct morphologies when imaged via QPI, with HCI-037 and HCI-038 being flatter while HCI-039 is more spindle-like (Fig. 2a). Despite differences in morphology, these three PDXO models have nearly the same average SGR (Fig. 2b) and single cell growth rate distributions (Fig. S5).

We measured the dose response behavior of HCI-037, HCI-038, and HCI-039 PDXO models (Fig. 2c, S5). We found that while all three PDXO models demonstrated a similar EC50 and DoR to doxorubicin (Fig. 2c), the models exhibit differential DoR in response to vinblastine (Fig. 2d) and birinapant (Fig. 2e), with the bone metastasis exhibiting a generally weaker DoR overall. This is evident in normalized mass data as well, with full response to birinapant resulting in residual growth at the end of the 48 h QPI assay (Figs. 2f, S6, S7).

### Multiparametric QPI characterizes heterogeneous growth behavior in PDXO model after selection for resistance to birinapant

**Fig. 3:**
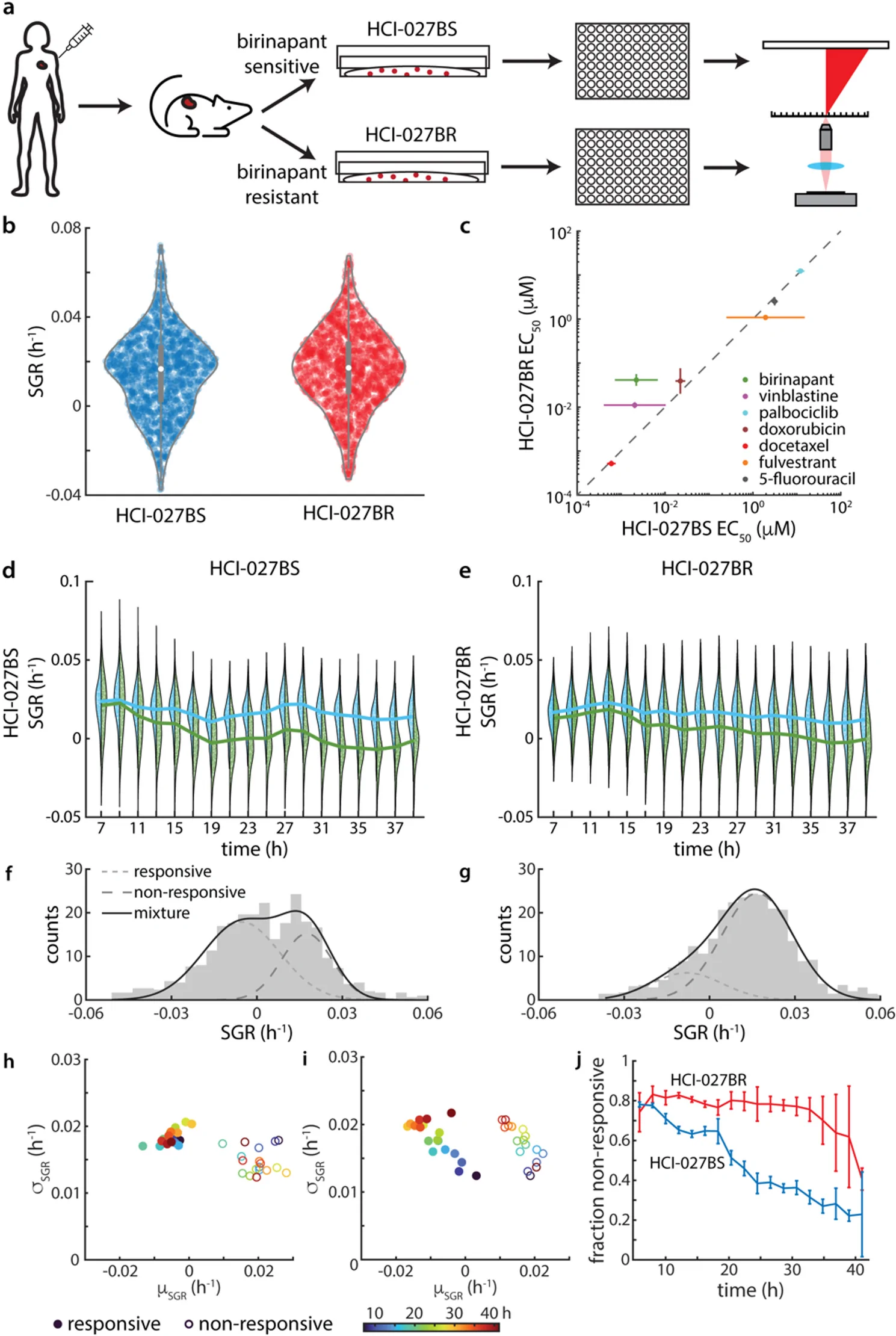
mQPI captures non-responsive subpopulation response in a heterogeneous sample. **a** Experiment workflow illustrating how models were derived and plated for QPI. **b** Violin plots of SGR for DMSO control (0.1%), *p* = 0.014 from a paired sample t-test. **c** Comparison of EC_50_ for HCI-027BR and HCI-027BS. **d**, **e** Side by side violin plots showing population SGR over time for (**d**) HCI-027BS and (**e**) HCI-027BR. At each timepoint, the left side distribution (blue) shows untreated cells and the right side distribution (green) shows cells treated with 1.6 nM birinapant. **f**, **g** Subpopulations distributions of SGR for (**f**) HCI-027BS and (**g**) HCI-027BR treated with 1.6 nM birinapant at 23 h. Subpopulations estimated from Gaussian mixture modeling shown as solid lines (small dash: responsive, large dash: non-responsive, black: mixture, R^2^ = 0.98 panel **f**, R^2^ = 0.99 panel **g**). **h**, **i** Time evolution of mean (*µ*) and standard deviation (*σ*) of responsive (closed symbols) subpopulations and non-responsive (open symbols) subpopulations from Gaussian mixture modeling in (**h**) HCI-027BS and (**i**) HCI-027BR. Symbols are colored by time (blue to red). **j** Non-responsive fraction of the population from the fitted model

We next tested two PDXO models that originate from the same tumor sample, with one cultured under standard culture conditions, HCI-027BS, and one cultured under selective pressure to enrich a population resistant to birinapant, HCI-027BR (Fig. 3a). As previously reported, these models show differential sensitivity to birinapant as PDXO [9, 59]. When grown in 2D culture for QPI, HCI-027BR grew slightly faster than the HCI-027BS (Fig. 3b). HCI-027BS also exhibited a notably more bimodal distribution than HCI-027BR (Fig. 3b).

EC_50_ measurements from the two models are strongly correlated to each other for most drugs (R^2^ = 0.916, *p* = 7 × 10^− 4^) (Fig. 3c, S8, S9). However, as expected, HCI-027BS has a significantly lower EC_50_ than HCI-027BR in response to birinapant (EC_50,HCI−027BS_ = 2.2 nM, EC_50,HCI−027BR_ = 41 nM). To evaluate if the difference in response to birinapant is due to the presence of a resistant subpopulation, we examined the distributions of single cell responses to birinapant over time (Fig. 3d, e). These data show significant heterogeneity in birinapant treated populations (Fig. S10), especially at intermediate timepoints, for example at 23 h (Fig. 3f, g). We fit these single cell SGR distributions of birinapant treated cells using a Gaussian mixture model. This model includes two populations, each modeled with a Gaussian distribution of SGR: a higher growth rate corresponding to a non-responsive population and lower growth rate corresponding to a responsive population. Surprisingly, in response to 1.6 nM birinapant (just below the EC50 for HCI-027BS), both HCI-027BS and HCI-027BR show subpopulations of responsive and non-responsive cells over time (Fig. 3h, i). Overall, the growth rate of the responsive populations in both HCI-027BS and HCI-027BR is negative, suggesting a cytotoxic effect on these cells. The non-responsive population from each model shows an initially high growth rate (~ 0.02 h^− 1^) indicating active growth. In HCI-027BS, both the responsive and non-responsive subpopulations show no clear trend in growth rate or distribution width (Fig. 3h). Additionally, the fraction of non-responsive cells in HCI-027BS decreases steadily over time (Fig. 3j). This suggests that cells in HCI-027BS show a delayed response, with no persistent, resistant subpopulation.

In contrast, in HCI-027BR, both responsive and non-responsive subpopulations show a trend towards decreasing growth rate, suggesting an impact in growth rate on both groups, with the responsive group becoming more acutely negative, indicating a strong cytotoxic effect, while the non-responsive group shows just a moderate decrease in growth rate, consistent with continued growth. However, the non-responsive subpopulation persists over the 45 h experiment (Fig. 3j). This suggests the presence of a dominant and persistent resistant population in HCI-027BR, as expected based on its derivation from a birinapant resistant PDX model. Overall, these data demonstrate the ability of QPI to identify drug resistant subpopulations from mixed samples.

### Rapid assessment of response via mQPI on high purity, direct-from-thaw patient samples

**Fig. 4:**
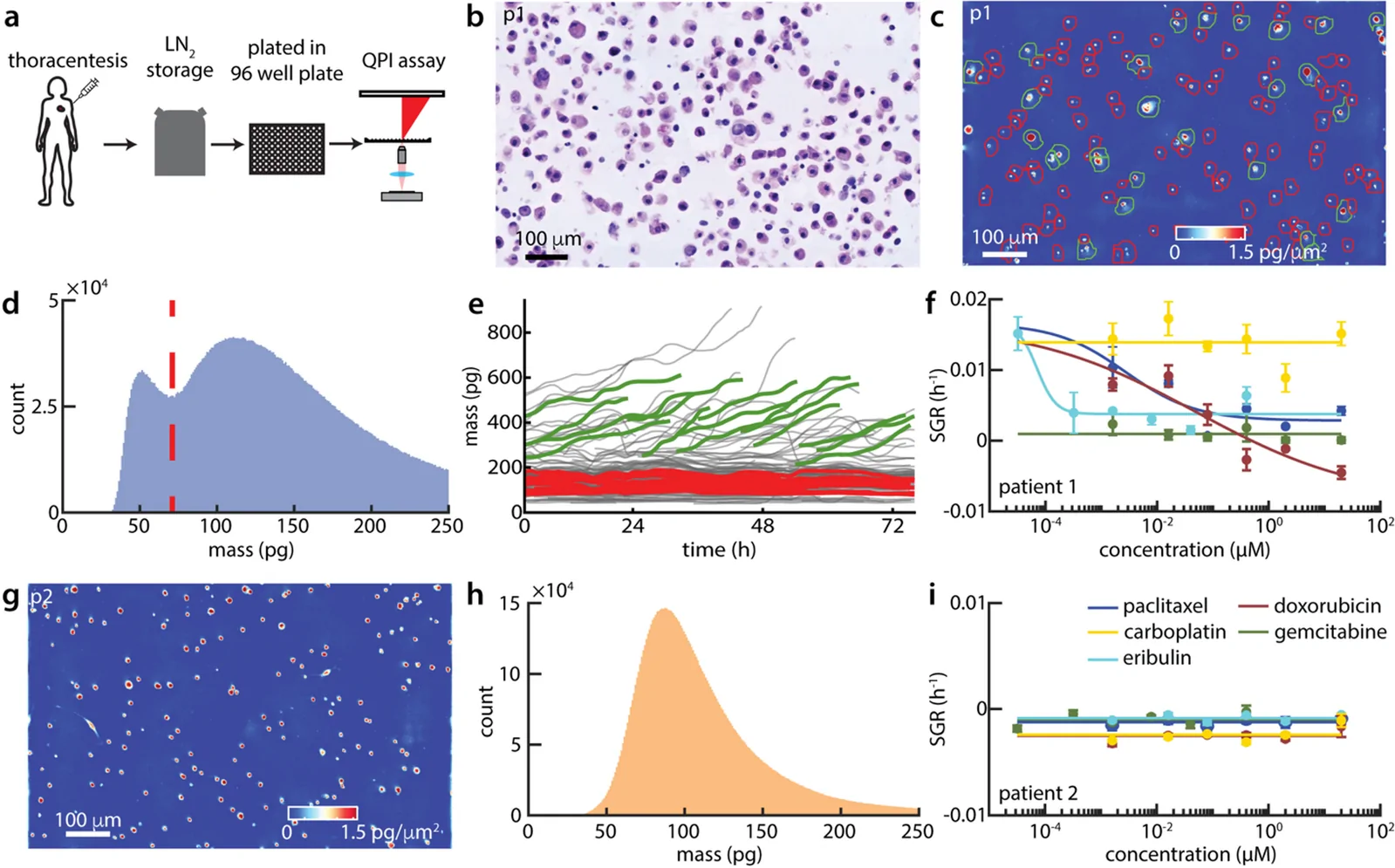
Direct-from-thaw patient samples analyzed using mQPI. **a** Patient sample workflow for QPI on patient pleural effusion samples direct-from-thaw after storage in liquid nitrogen (LN_2_). **b** H&E stain image for pleural effusion sample from patient 1 (p1). **c** QPI image for patient 1 cells from small cell enriched region. Green/red segmentation outlines indicate cells greater/less than the mass threshold shown in panel d. **d** Distribution of cell mass across all positions for patient 1. Red dashed line is a mass threshold. **e** Single cell mass over time for one well of vehicle (0.1% DMSO) control from patient (1) Select cells above/below the mass threshold are highlighted in green/red, respectively. **f** Drug response for patient 1, direct-from-thaw. **g** Representative QPI image of patient 2 cells. **h** Distribution of cell mass across all positions for patient (2). **i **Drug response for patient 2, direct from thaw

Two pleural effusion samples, procured as fresh frozen and banked at Huntsman Cancer Institute, were tested by mQPI. Samples were thawed from liquid nitrogen storage and depleted of CD45 + cells via magnetic bead sorting to enrich for non-immune cells including tumor cells. We then plated the primary cells in 2D monolayer cultured in Clevers’ complete medium [60] in a 96 well plate for 18 h prior to drug treatments [9, 59, 69] (Fig. 4a). Both samples were characterized via Hematoxylin and Eosin (H&E) staining (Fig. 4b) and immunohistochemistry (Figs. S11, S12) to confirm the presence of cells with tumor-like morphology and staining (Fig. 4b). When imaged using QPI the sample from patient 1 displayed two distinct cell sizes (Fig. 4c) and the overall mass distribution of single cells displays two distinct peaks (Fig. 4d). We used that distinction in mass apparent by the bimodal distribution in patient 1 (Fig. 4d) to filter out the smaller cells that did not grow in control conditions and likely represented residual immune and/or other non-tumor cells (Fig. 4e). This resulted in a clearly discernable drug response among the larger mass cells (Fig. 4f). The sample from patient 2, however, had cells that were more uniform in size, as observed in QPI images (Fig. 4g) and single cell mass distributions (Fig. 4h). This sample also had notably higher positive staining for CD45 (Fig. S12). Furthermore, we were unable to identify a subpopulation of cells with a differential response in the patient 2 direct-from-thaw sample, with all dosed conditions (Fig. 4i) and untreated control (SGR_patient2,DMSO_ = -2.2 × 10^− 3^ h^− 1^) both having a slightly negative SGR.

### Short expansion of primary cells enriches tumor population enabling analysis by mQPI

**Fig. 5:**
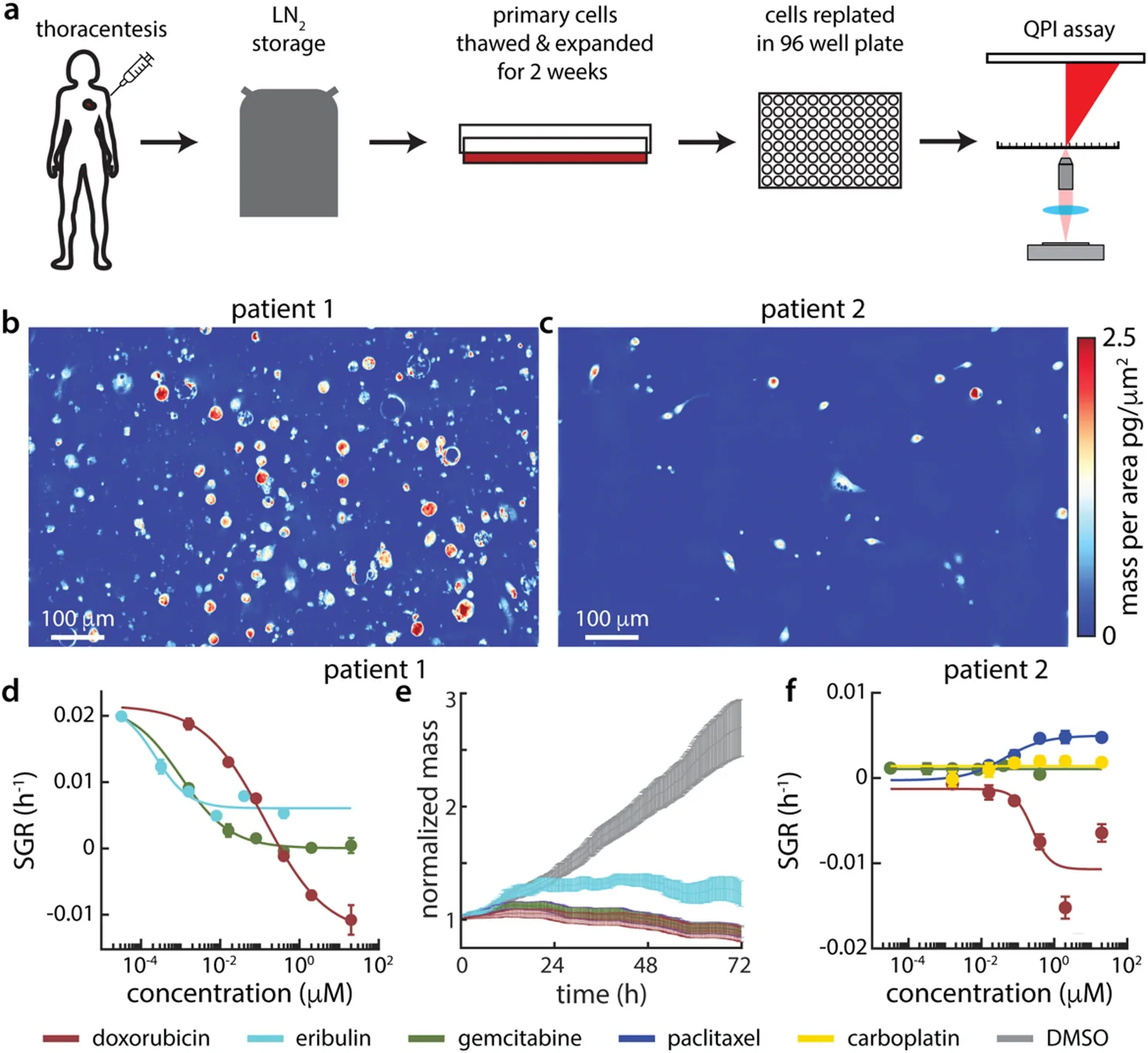
Short expansion enriches cell population for functional drug testing by mQPI. **a** Workflow for pleural effusion patient sample expansion prior to QPI imaging. **b** QPI image of patient 1 cells after expansion. **c** Phase image for patient 2 cells after expansion. **d** Dose response for patient 1 after expansion. **e** Normalized mass over time for patient 1 cells after expansion (doxorubicin: 400 nM; gemcitabine: 400 nM; eribulin: 1.6 nM, DMSO: 0.1%). **f** Dose response for patient 2 after expansion. Colors corresponding to drugs for (**e**-**f**) indicated at the bottom of the figure

To allow cells to acclimate after thawing and to enrich the subpopulation of growing cells (Fig. 4e), we tested the effect of a short-term expansion by culturing primary cells in 2D with regular media changes for two weeks (Fig. 5a). This successfully increased the fraction of large, growing cells in both patient pleural effusion samples (Fig. 5b, c). Patient 1 drug response were consistent with results in the direct-from-thaw experiment (compare Figs. 4f and 5d), and visible in normalized mass over time as well (Fig. 5e). Similar to the maintenance of drug sensitivities observed for expansion of PDO in Matrigel [59], these responses were also maintained in 2D culture on PEI and Matrigel for at least 6 weeks (Fig. S14, S15). For patient 2, we were able to gauge a response after short-term 2D expansion that we were unable to observe in the direct-from-thaw setting, including doxorubicin sensitivity and mild stimulatory effects of paclitaxel (Fig. 5f). Overall, this proof of concept suggests that mQPI analysis can be applied to a broader range of patient samples with a short-term expansion period.

## Discussion

We demonstrated that mQPI can provide functional cell data for PDMCs, including both PDXO cells and cells from viably frozen patient samples. For PDXOs originating from the same patient, mQPI could identify different drug sensitivity profiles based on anatomic site and/or time of collection (Fig. 2), which underscores the importance of accounting for site-specific tumor heterogeneity when selecting therapies. The ability of mQPI to track single cells also allows for heterogeneity to be directly observed. In application to a sample with mixed responses (Fig. 3), mQPI was able to distinguish resistant subpopulations, in contrast to conventional, bulk assays, which would be unable to resolve single cell responses. Additionally, as in previous work [55], mQPI measurements (ToR, DoR, EC50, SD) had low correlation to one another, indicating that we were able to capture independent measurements of response to therapy in PDMCs (Fig. S15).

We also demonstrated the ability of mQPI to detect response from viably frozen patient samples, which could represent a key step toward practical application of mQPI in the clinical setting. Our overall assay time was either days (direct-from-thaw, Fig. 4) or 2.5 weeks (Fig. 5), which is a significant improvement over the months often required to establish PDX or PDO models. Our data indicate mQPI is able to capture a response from a very small subset of cells, addressing a key limitation of functional assays in clinical settings. It is worth noting that for this study, we plated approximately 500,000 cells per experimental condition, but performed imaging on approximately 50,000 cells. This represents just a 10% sample utilization, which can likely be further improved with optimized sample handling.

A key limitation of the current approach is that patient samples remained contaminated with non-tumor cell types even after CD45 depletion, requiring computational enrichment of the tumor population. Our mass-based filter increased the percentage of tumor cells evaluated and enabled a tumor cell response to be gauged in mixed cultures, but it relies strictly on a discernible mass difference between tumor and non-tumor cells. This distinction was clear for Patient 1, whose sample showed a bimodal mass distribution (Fig. 4d), but was not sufficient for Patient 2, whose cells were more uniform in size (Fig. 4h) and showed notably higher CD45 + staining (Fig. S12). Consequently, mass-based filtering alone may fail in samples with uniform size distributions or high immune or stromal contamination, and in such cases a short-term (2-week) expansion may be required to enrich the viable tumor population, as we observed for Patient 2 (Fig. 5). Future work will examine alternative filtering methods, including label-free and machine learning/AI-assisted approaches based on cell morphology or other features accessible via QPI.

In future translational application, mQPI can potentially be used to complement other precision medicine approaches. For example, dynamic BH3 profiling (DBP) measures early drug-induced apoptotic priming in patient-derived cells [11, 12]. In contrast to mQPI, which quantifies integrated phenotypic responses over time, DBP focuses on a pathway-proximal apoptotic signal measured shortly after drug exposure. As a result, DBP is particularly informative for therapies whose dominant mechanism of action involves rapid induction of apoptosis, whereas mQPI captures a broader range of response phenotypes, including cytostatic effects, delayed responses, and heterogeneous growth trajectories. Organoid-based drug response assays, including PDXO platforms, have demonstrated the value of patient-derived models for functional testing and are commonly implemented using endpoint viability measurements such as CellTiter-Glo [9, 59, 60]. While these assays provide scalable estimates of drug sensitivity, endpoint metabolic readouts necessarily average responses across populations and time, limiting their ability to resolve response kinetics or cell-to-cell heterogeneity. In this context, mQPI provides complementary information by measuring longitudinal single-cell trajectories, enabling direct quantification of response dynamics and identification of resistant or slowly responding subpopulations that may be obscured in bulk endpoint assays. Additionally, molecular profiling remains central to precision oncology, yet clinical experience has highlighted limitations in predicting therapeutic response from genomic alterations alone [1]. These limitations have motivated increased interest in functional precision oncology approaches that empirically assess drug response in patient-derived material [5–8]. Our findings support a complementary model in which genomic profiling is used to nominate candidate therapies or mechanisms, while mQPI provides a direct phenotypic assessment of drug sensitivity, heterogeneity, and resistance-associated behavior on clinically relevant time scales. Such integration may be particularly valuable for capturing non-genetic determinants of response that are not readily inferred from baseline molecular data. Finally, while this study used a custom DPC QPI microscope, the multiparametric analysis presented here is not tied to a specific QPI instrument. Quantitative phase can be recovered on standard, commercially available microscopes using computational approaches such as the transport-of-intensity equation [26, 27], or on commercially available, dedicated QPI platforms such as PhaseFocus LiveCyte [70, 71] and PHI Holomonitor [72]. DPC itself operates on an otherwise standard microscope, indicating that this analysis could be translated to more widely accessible imaging platforms.

## Conclusion

In summary, mQPI is a rapid, accessible, and robust tool for analyzing cancer cell responses to therapies, offering insights into growth, heterogeneity, and drug sensitivity. To enhance clinical relevance, future efforts should focus on optimizing tumor cell enrichment, whether through improvements in sample preparation or through computational filtering, and should be extended to comparison with known clinical outcomes in larger sample cohorts. The ability to rapidly assess therapeutic responses using direct-from-thaw samples or after short-term expansions underscores its potential as a time-efficient alternative to current models. Ultimately, mQPI holds promise for advancing functional precision oncology and improving personalized treatment strategies.

## Supplementary Information

Supplementary Material 1.

## Data Availability

Data is provided within the manuscript or supplementary information files.

## References

[1] Flaherty KT, Gray RJ, Chen AP, Li S, McShane LM, Patton D, et al. Molecular Landscape and Actionable Alterations in a Genomically Guided Cancer Clinical Trial: National Cancer Institute Molecular Analysis for Therapy Choice (NCI-MATCH). J Clin Oncol. 2020;38(33):3883–94. 10.1200/JCO.19.03010 PMC7676882 33048619

[2] Milbury CA, Creeden J, Yip WK, Smith DL, Pattani V, Maxwell K, et al. Clinical and analytical validation of FoundationOne^®^CDx, a comprehensive genomic profiling assay for solid tumors. PLoS ONE. 2022;17(3):e0264138. 10.1371/journal.pone.0264138 PMC8926248 35294956

[3] Schettini F, Sirico M, Loddo M, Williams GH, Hardisty KM, Scorer P, et al. Next-generation sequencing-based evaluation of the actionable landscape of genomic alterations in solid tumors: the MOZART prospective observational study. Oncologist. 2025;30(1)oyae206. 10.1093/oncolo/oyae206. PMC11783315 39177668

[4] Vander Velde R, Yoon N, Marusyk V, Durmaz A, Dhawan A, Miroshnychenko D, et al. Resistance to targeted therapies as a multifactorial, gradual adaptation to inhibitor specific selective pressures. Nat Commun. 2020;11(1):2393. 10.1038/s41467-020-16212-w PMC7224215 32409712

[5] Letai A. Functional precision cancer medicine-moving beyond pure genomics. Nat Med. 2017;23(9):1028–35. 10.1038/nm.4389 28886003

[6] Letai A, Bhola P, Welm AL. Functional precision oncology: Testing tumors with drugs to identify vulnerabilities and novel combinations. Cancer Cell. 2022;40(1):26–35. 10.1016/j.ccell.2021.12.004 PMC8752507 34951956

[7] Friedman AA, Letai A, Fisher DE, Flaherty KT. Precision medicine for cancer with next-generation functional diagnostics. Nat Rev Cancer. 2015;15(12):747–56. 10.1038/nrc4015 PMC4970460 26536825

[8] Blanchard Z, Brown EA, Ghazaryan A, Welm AL. PDX models for functional precision oncology and discovery science. Nat Rev Cancer. 2025;25(3):153–66. 10.1038/s41568-024-00779-3 PMC12124142 39681638

[9] Guillen KP, Fujita M, Butterfield AJ, Scherer SD, Bailey MH, Chu Z, et al. A human breast cancer-derived xenograft and organoid platform for drug discovery and precision oncology. Nat Cancer. 2022;3(2):232–50. 10.1038/s43018-022-00337-6 PMC8882468 35221336

[10] Ding S, Hsu C, Wang Z, Natesh NR, Millen R, Negrete M, et al. Patient-derived micro-organospheres enable clinical precision oncology. Cell Stem Cell. 2022;29(6):905–17. e6. 10.1016/j.stem.2022.04.006 PMC9177814 35508177

[11] Montero J, Sarosiek KA, DeAngelo JD, Maertens O, Ryan J, Ercan D, et al. Drug-induced death signaling strategy rapidly predicts cancer response to chemotherapy. Cell. 2015;160(5):977–89. 10.1016/j.cell.2015.01.042 PMC4391197 25723171

[12] Bhola PD, Ahmed E, Guerriero JL, Sicinska E, Su E, Lavrova E, et al. High-throughput dynamic BH3 profiling may quickly and accurately predict effective therapies in solid tumors. Sci Signal. 2020;13:636. 10.1126/scisignal.aay1451 PMC8023011 32546544

[13] Stockslager MA, Malinowski S, Touat M, Yoon JC, Geduldig J, Mirza M, et al. Functional drug susceptibility testing using single-cell mass predicts treatment outcome in patient-derived cancer neurosphere models. Cell Rep. 2021;37(1):109788. 10.1016/j.celrep.2021.109788 34610309

[14] Kimmerling RJ, Stevens MM, Olcum S, Minnah A, Vacha M, LaBella R, et al. A pipeline for malignancy and therapy agnostic assessment of cancer drug response using cell mass measurements. Commun Biology. 2022;5(1):1295. 10.1038/s42003-022-04270-3 PMC9701192 36435843

[15] Liu Y, Uttam S. Perspective on quantitative phase imaging to improve precision cancer medicine. J Biomed Opt. 2024;29(Suppl 2):S22705. 10.1117/1.JBO.29.S2.S22705 PMC10996848 38584967

[16] Popescu G, Park K, Mir M, Bashir R. New technologies for measuring single cell mass. Lab Chip. 2014;14(4):646–52. 10.1039/c3lc51033f 24322181

[17] Zangle TA, Teitell MA, Reed J. Live cell interferometry quantifies dynamics of biomass partitioning during cytokinesis. PLoS ONE. 2014;9(12)e115726. 10.1371/journal.pone.0115726. PMC4274116 25531652

[18] Popescu G, Park Y, Lue N, Best-Popescu C, Deflores L, Dasari RR, et al. Optical imaging of cell mass and growth dynamics. Am J Physiol-Cell Physiol. 2008;295(2):C538–44. 10.1152/ajpcell.00121.2008 PMC2518415 18562484

[19] Ettinger A, Wittmann T. Fluorescence live cell imaging. Methods Cell Biol. 2014;123:77–94. 10.1016/B978-0-12-420138-5.00005-7 PMC4198327 24974023

[20] Mitchison J, Passano L, Smith F. An integration method for the interference microscope. J Cell Sci. 1956;3(38):287–302.

[21] Mitchison JM. The growth of single cells: I. Schizosaccharomyces pombe. Exp Cell Res. 1957;13(2):244–62. 10.1016/0014-4827(57)90005-8 13480293

[22] Horstmeyer R, Chung J, Ou X, Zheng G, Yang C. Diffraction tomography with Fourier ptychography. Optica. 2016;3(8):827–35. 10.1364/OPTICA.3.000827 PMC5521281 28736737

[23] Cuche E, Bevilacqua F, Depeursinge C. Digital holography for quantitative phase-contrast imaging. Opt Lett. 1999;24(5):291–3. 10.1364/ol.24.000291 18071483

[24] Primot J, Sogno L. Achromatic three-wave (or more) lateral shearing interferometer. J Opt Soc Am A. 1995;12(12):2679–85.

[25] Chaumet PC, Bon P, Maire G, Sentenac A, Baffou G. Quantitative phase microscopies: accuracy comparison. Light Sci Appl. 2024;13(1):288. 10.1038/s41377-024-01619-7 PMC11470049 39394163

[26] Grant SD, Richford K, Burdett HL, McKee D, Patton BR. Low-cost, open-access quantitative phase imaging of algal cells using the transport of intensity equation. Royal Soc Open Sci. 2020;7(1):191921. 10.1098/rsos.191921. PMC7029887 32218984

[27] Ma H, Chen M, Xu J, Yang Y, Zhao Y, Liu Y. An Omni-Mesoscope for multiscale high-throughput quantitative phase imaging of cellular dynamics and high-content molecular characterization. Sci Adv. 2024;10:eadq5009. 10.1126/sciadv.adq5009 PMC11482309 39413179

[28] Baffou G. Wavefront Microscopy Using Quadriwave Lateral Shearing Interferometry: From Bioimaging to Nanophotonics. ACS Photonics. 2023;10(2):322–39.

[29] Bon P, Maucort G, Wattellier B, Monneret S. Quadriwave lateral shearing interferometry for quantitative phase microscopy of living cells. Opt Express. 2009;17(15):13080–94. 10.1364/oe.17.013080 19654713

[30] Aknoun S, Yonnet M, Djabari Z, Graslin F, Taylor M, Pourcher T, et al. Quantitative phase microscopy for non-invasive live cell population monitoring. Sci Rep. 2021;11(1):4409. 10.1038/s41598-021-83537-x PMC7904828 33627679

[31] Zheng C, Wang T, Li Z, Sun R, Yang D, Wang S, et al. Quantitative phase imaging based on fourier ptychographic microscopy: advances, applications, and perspectives. Adv Imaging. 2025;2(3):032001. 10.3788/AI.2025.20001.

[32] Zheng G, Horstmeyer R, Yang C. Wide-field, high-resolution Fourier ptychographic microscopy. Nat Photonics. 2013;7(9):739–45. 10.1038/nphoton.2013.187 PMC4169052 25243016

[33] Marquet P, Rappaz B, Magistretti PJ, Cuche E, Emery Y, Colomb T, et al. Digital holographic microscopy: A noninvasive contrastimaging technique allowing quantitative visualization ofliving cells with subwavelength axial accuracy. Opt Lett. 2005;30(5):468–70. 10.1364/ol.30.000468 15789705

[34] Javidi B, Carnicer A, Anand A, Barbastathis G, Chen W, Ferraro P, et al. Roadmap on digital holography [Invited]. Opt Express. 2021;29:22. 10.1364/OE.435915 34808951

[35] Pavillon N, Kühn J, Moratal C, Jourdain P, Depeursinge C, Magistretti PJ, et al. Early cell death detection with digital holographic microscopy. PLoS ONE. 2012;7(1):e30912. 10.1371/journal.pone.0030912 PMC3269420 22303471

[36] Tian L, Liu Z, Yeh L-H, Chen M, Zhong J, Waller L. Computational illumination for high-speed in vitro Fourier ptychographic microscopy. Optica. 2015;2(10):904.

[37] Liu Z, Tian L, Liu S, Waller L. Real-time brightfield, darkfield, and phase contrast imaging in a light-emitting diode array microscope. J Biomed Opt. 2014;19(10):106002. 10.1117/1.JBO.19.10.106002 25271540

[38] Barker KL, Boucher KM, Judson-Torres RL. Label-Free Classification of Apoptosis, Ferroptosis and Necroptosis Using Digital Holographic Cytometry. Appl Sci. 2020;10(13):4439.

[39] McNeal AS, Belote RL, Zeng H, Urquijo M, Barker K, Torres R, et al. BRAFV600E induces reversible mitotic arrest in human melanocytes via microRNA-mediated suppression of AURKB. eLife. 2021;10:e70385. 10.7554/eLife.70385 PMC8610417 34812139

[40] Vicar T, Raudenska M, Gumulec J, Balvan J. The Quantitative-Phase Dynamics of Apoptosis and Lytic Cell Death. Sci Rep. 2020;10(1):1566. 10.1038/s41598-020-58474-w PMC6994697 32005874

[41] Pradeep S, Zangle TA. Quantitative phase velocimetry measures bulk intracellular transport of cell mass during the cell cycle. Sci Rep. 2022;12(1):6074. 10.1038/s41598-022-10000-w PMC9005622 35414087

[42] Kandel ME, Kim E, Lee YJ, Tracy G, Chung HJ, Popescu G. Multiscale assay of unlabeled neurite dynamics using phase imaging with computational specificity. ACS Sens. 2021;6(5):1864–74. 10.1021/acssensors.1c00100. PMC8815662 33882232

[43] Eldridge WJ, Ceballos S, Shah T, Park HS, Steelman ZA, Zauscher S, et al. Shear Modulus Measurement by Quantitative Phase Imaging and Correlation with Atomic Force Microscopy. Biophys J. 2019;117(4):696–705. 10.1016/j.bpj.2019.07.008 PMC6712492 31349989

[44] Nguyen TL, Polanco ER, Patananan AN, Zangle TA, Teitell MA. Cell viscoelasticity is linked to fluctuations in cell biomass distributions. Sci Rep. 2020;10(1):11. 10.1038/s41598-020-64259-y PMC7198624 32366921

[45] Vicar T, Chmelik J, Navratil J, Kolar R, Chmelikova L, Cmiel V, et al. Cancer cell viscoelasticity measurement by quantitative phase and flow stress induction. Biophys J. 2022;121(9):1632–42. 10.1016/j.bpj.2022.04.002. PMC9117928 35390297

[46] Pradeep S, Tasnim T, Zhang H, Zangle TA. Simultaneous measurement of neurite and neural body mass accumulation via quantitative phase imaging. Analyst. 2021;146(4):1361–8. 10.1039/d0an01961e. 33393564

[47] Haouat M, Lariviere-Loiselle C, Crochetiere ME, Chaniot J, Moreaud M, Belanger E, et al. Visualizing the fine structure and dynamics of living cells with temporal polychromatic digital holographic microscopy. J Opt Soc Am Opt Image Sci Vis. 2024;41(11):C109–24. 10.1364/JOSAA.534150 39889082

[48] Hu C, Sam R, Shan M, Nastasa V, Wang M, Kim T, et al. Optical excitation and detection of neuronal activity. J Biophotonics. 2019;12(3):e201800269. 10.1002/jbio.201800269 30311744

[49] Uttam S, Pham HV, LaFace J, Leibowitz B, Yu J, Brand RE, et al. Early Prediction of Cancer Progression by Depth-Resolved Nanoscale Mapping of Nuclear Architecture from Unstained Tissue Specimens. Cancer Res. 2015;75(22):4718–27. 10.1158/0008-5472.CAN-15-1274 PMC4651746 26383164

[50] Uttam S, Hashash JG, LaFace J, Binion D, Regueiro M, Hartman DJ, et al. Three-Dimensional Nanoscale Nuclear Architecture Mapping of Rectal Biopsies Detects Colorectal Neoplasia in Patients with Inflammatory Bowel Disease. Cancer Prev Res (Phila). 2019;12(8):527–38. 10.1158/1940-6207.CAPR-19-0024 PMC6677599 31164345

[51] Bokemeyer A, Buskermolen J, Ketelhut S, Tepasse PR, Vollenberg R, Trebicka J, et al. Quantitative phase imaging using digital holographic microscopy to assess the degree of intestinal inflammation in patients with ulcerative colitis. J Clin Med. 2023;12(12):4067. 10.3390/jcm12124067. PMC10299047 37373760

[52] de Haan K, Zhang Y, Zuckerman JE, Liu T, Sisk AE, Diaz MFP, et al. Deep learning-based transformation of H&E stained tissues into special stains. Nat Commun. 2021;12(1):4884. 10.1038/s41467-021-25221-2 PMC8361203 34385460

[53] Park J, Bai B, Ryu D, Liu T, Lee C, Luo Y, et al. Artificial intelligence-enabled quantitative phase imaging methods for life sciences. Nat Methods. 2023;20(11):1645–60. 10.1038/s41592-023-02041-4 37872244

[54] Mir M, Bergamaschi A, Katzenellenbogen BS, Popescu G. Highly sensitive quantitative imaging for monitoring single cancer cell growth kinetics and drug response. PLoS ONE. 2014;9(2):e89000. 10.1371/journal.pone.0089000 PMC3928317 24558461

[55] Polanco ER, Moustafa TE, Butterfield AJ, Scherer SD, Cortes-Sanchez E, Bodily T, et al. Multiparametric quantitative phase imaging for real-time, single cell, drug screening in breast cancer. Commun Biology. 2022;5:794. 10.1038/s42003-022-03759-1 PMC9360018 35941353

[56] Huang D, Roy IJ, Murray GF, Reed J, Zangle TA, Teitell MA. Identifying fates of cancer cells exposed to mitotic inhibitors by quantitative phase imaging. Analyst. 2019;145(1):97–106. 10.1039/c9an01346f. PMC6917840 31746831

[57] Reed J, Chun J, Zangle TA, Kalim S, Hong JS, Pefley SE, et al. Rapid, massively parallel single-cell drug response measurements via live cell interferometry. Biophys J. 2011;101(5):1025–31. 10.1016/j.bpj.2011.07.022 PMC3164181 21889438

[58] Tebon PJ, Wang B, Markowitz AL, Davarifar A, Tsai BL, Krawczuk P, et al. Drug screening at single-organoid resolution via bioprinting and interferometry. Nat Commun. 2023;14(1):3168. 10.1038/s41467-023-38832-8 PMC10244450 37280220

[59] Scherer SD, Zhao L, Butterfield AJ, Yang CH, Cortes-Sanchez E, Guillen KP, et al. Breast cancer PDxO cultures for drug discovery and functional precision oncology. STAR Protoc. 2023;4(3):102402. 10.1016/j.xpro.2023.102402 PMC10339058 37402170

[60] Sachs N, de Ligt J, Kopper O, Gogola E, Bounova G, Weeber F, et al. A Living Biobank of Breast Cancer Organoids Captures Disease Heterogeneity. Cell. 2018;172(1–2):373–e8610. 10.1016/j.cell.2017.11.010 29224780

[61] Tian L, Waller L. Quantitative differential phase contrast imaging in an LED array microscope. Opt Express. 2015;23(9):11394–403. 10.1364/OE.23.011394 25969234

[62] Zangle TA, Teitell MA. Live-cell mass profiling: an emerging approach in quantitative biophysics. Nat Methods. 2014;11(12):1221–8. 10.1038/nmeth.3175 PMC4319180 25423019

[63] Crocker JC, Grier DG. Methods of Digital Video Microscopy for Colloidal Studies. J Colloid Interface Sci. 1996;179:298–310.

[64] Kitsos C, Toulias T. Hellinger Distance Between Generalized Normal Distributions. Br J Math Comput Sci. 2017;21(2):1–16.

[65] Lin LI. A concordance correlation coefficient to evaluate reproducibility. Biometrics. 1989;45(1):255–68. 2720055

[66] Rüegg UT, Hefti F. Growth of dissociated neurons in culture dishes coated with synthetic polymeric amines. Neurosci Lett. 1984;49(3):319–24. 10.1016/0304-3940(84)90309-4 6493615

[67] DeRose YS, Gligorich KM, Wang G, Georgelas A, Bowman P, Courdy SJ, et al. Patient-derived models of human breast cancer: protocols for in vitro and in vivo applications in tumor biology and translational medicine. Curr Protoc Pharmacol. 2013;Chap 14:Unit1423. 10.1002/0471141755.ph1423s60 PMC3630511 23456611

[68] Mu L, Kang JH, Olcum S, Payer KR, Calistri NL, Kimmerling RJ, et al. Mass measurements during lymphocytic leukemia cell polyploidization decouple cell cycle- and cell size-dependent growth. Proc Natl Acad Sci USA. 2020;117(27):15659–65. 10.1073/pnas.1922197117 PMC7355023 32581119

[69] Wang Z, Boretto M, Millen R, Natesh N, Reckzeh ES, Hsu C, et al. Rapid tissue prototyping with micro-organospheres. Stem Cell Rep. 2022;17(9):1959–75. 10.1016/j.stemcr.2022.07.016 PMC9481922 35985334

[70] Godden TM, Suman R, Humphry MJ, Rodenburg JM, Maiden AM. Ptychographic microscope for three-dimensional imaging. Opt Express. 2014;22(10):12513–23. 10.1364/OE.22.012513 24921369

[71] Zhang Y, Urquijo MA, Zitnay RG, Marks K, Belote RL, Hansen MMK, et al. A BRN2:MYC transcriptional axis regulates interconversion between therapy-resistant and tumorigenic phenotypes in melanoma. Cell Rep. 2025;44(12):116675. 10.1016/j.celrep.2025.116675 PMC12834598 41405996

[72] Janicke B, Karsnas A, Egelberg P, Alm K. Label-free high temporal resolution assessment of cell proliferation using digital holographic microscopy. Cytometry A. 2017;91(5):460–9. 10.1002/cyto.a.23108 28437571

